# P-2176. Validation of Air Sampling and Real-Time Quantitative Polymerase Chain Reaction for Measles Virus Surveillance: A Single-Center, Preliminary Assessment

**DOI:** 10.1093/ofid/ofae631.2330

**Published:** 2025-01-29

**Authors:** Marielle J Fricchione, Colleen B Nash, David Nguyen, Hannah Barbian, Mary K Hayden

**Affiliations:** Rush University Medical Center, Chicago, Illinois; Rush University Medical Center, Chicago, Illinois; Rush University Medical Center, Chicago, Illinois; Rush University Medical Center, Regional Innovative Public Health Laboratory, Chicago, Illinois; Rush University Medical Center, Chicago, Illinois

## Abstract

**Background:**

During the early months of Chicago’s 2024 measles outbreak, many patients were admitted to local hospitals due to a lack of isolation housing in congregate settings. While the infection control potential of air sampling for viral pathogens is well described for viruses such as SARS-CoV-2, its utility for measles virus response is less clear. Here, we report a single center assessment of inpatient air sampling from airborne isolation rooms of patients with and without PCR-confirmed measles infection.

**Figure.**
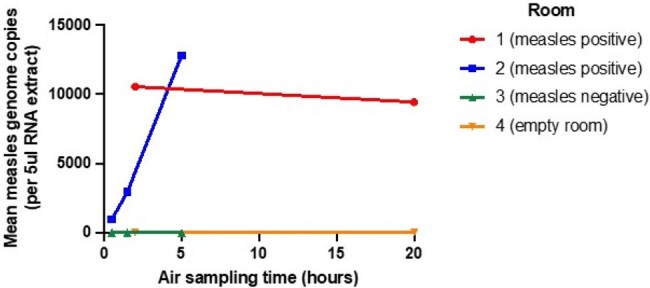
Mean Measles Genome Copies in Four Rooms by Air Sampling Time

**Methods:**

Aerosolsense samplers (ThermoFisher) were placed in 3 occupied and 1 unoccupied (negative control) airborne isolation rooms under negative pressure conditions. Air samples were collected at varying timeframes (30 minutes – 20 hours). Total nucleic acids were extracted from air samples within 24 hours of collection. Quantitative reverse transcription PCR (qRT-PCR) was performed to detect wildtype measles virus and genotype A (vaccine strain) measles virus; a gBlock DNA standard was included to estimate viral copy numbers. N450 amplification and Sanger sequencing were performed on selected positive samples to determine measles genotype.

**Table:**
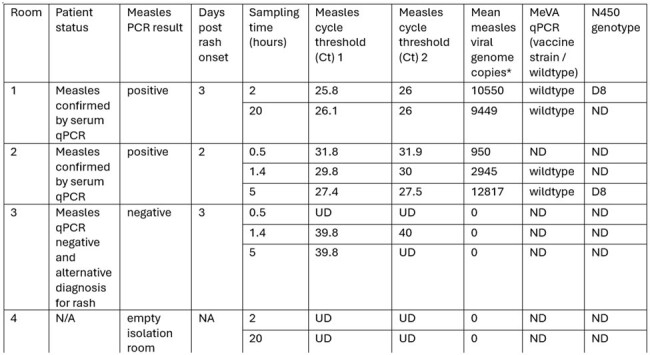
Inpatient Measles Air Sampling Data at Different Time Intervals *per 5 μl cartridge nucleic acid extract, viral copies approximated using measles N1 DNA gBlock, ND: not done, UD: undetermined, NA: not applicable

**Results:**

Air sampling methods were successful, with measles PCR positivity only in rooms with a measles. Measles patients were 2-3 days post rash onset. The air samples collected between 0.5 – 5 hours showed linearly-increasing measles viral load from 950-10,550 genome copies. [Figure] Samples collected at 20 hours did not show increased virus load compared to 2 hours, with mean 10,000 viral copies, indicating possible saturation over extended air sampling. Sequences derived from air samples were determined to be wildtype measles virus genotype D8 (the circulating genotype in Chicago at the time). [Table]

**Conclusion:**

Air sampling in patient rooms allowed genotyping of measles virus in real-time and shows promise for describing the dynamics of measles viral load and potential for transmissibility. The utility of air sampling as a tool for measles surveillance and outbreak management warrants further testing in healthcare and congregate settings.

**Disclosures:**

All Authors: No reported disclosures

